# The connection domain in reverse transcriptase facilitates the *in vivo *annealing of tRNA^Lys3 ^to HIV-1 genomic RNA

**DOI:** 10.1186/1742-4690-1-33

**Published:** 2004-10-19

**Authors:** Shan Cen, Meijuan Niu, Lawrence Kleiman

**Affiliations:** 1 Lady Davis Institute for Medical Research and McGill AIDS Centre, Jewish General Hospital, Montreal, Quebec, Canada H3T 1E2; 2 Department of Medicine, McGill University, Montreal, Quebec, Canada H3T 1E2; 3 Department of Microbiology and Immunology, McGill University, Montreal, Quebec, Canada H3T 1E2

## Abstract

The primer tRNA for reverse transcription in HIV-1, tRNA^Lys3^, is selectively packaged into the virus during its assembly, and annealed to the viral genomic RNA. The ribonucleoprotein complex that is involved in the packaging and annealing of tRNA^Lys ^into HIV-1 consists of Gag, GagPol, tRNA^Lys^, lysyl-tRNA synthetase (LysRS), and viral genomic RNA. Gag targets tRNA^Lys ^for viral packaging through Gag's interaction with LysRS, a tRNA^Lys^-binding protein, while reverse transcriptase (RT) sequences within GagPol (the thumb domain) bind to tRNA^Lys^. The further annealing of tRNA^Lys3 ^to viral RNA requires nucleocapsid (NC) sequences in Gag, but not the NC sequences GagPol. In this report, we further show that while the RT connection domain in GagPol is not required for tRNA^Lys3 ^packaging into the virus, it is required for tRNA^Lys3 ^annealing to the viral RNA genome.

## Background

During assembly of HIV-1, the major tRNA^Lys ^isoacceptors in mammalian cells, tRNA^Lys1,2 ^and tRNA^Lys3^, are selectively incorporated into the virus [1]. tRNA^Lys3 ^is the primer for initiating minus-strand cDNA synthesis, and its annealing to the 18 nucleotide primer binding site (PBS) region in the 5' part of the viral genome via the 3' 18 nucleotides in tRNA^Lys3 ^complementary to the PBS, is a key step in viral replication [2]. Other regions upstream and downstream of the PBS may also anneal with additional sequences in the tRNA [3,4].

Both tRNA^Lys3 ^and sites of annealing in viral RNA contain double stranded regions which may require denaturation for annealing to proceed efficiently. Nucleocapsid protein (NC) has been shown to facilitate tRNA^Lys3 ^annealing both *in vitro *[5,6] and *in vivo *[7], primarily through basic amino acids flanking the first zinc finger. While NC may destabilize viral RNA secondary structure, it has been demonstrated by several groups that nucleocapsid protein does not unwind the secondary structure of tRNA *in vitro*, and that the protein only has very subtle tertiary structural and helix destabilization effects on tRNA^Lys3 ^alone [8-11].

Although processed nucleocapsid proteins have been shown to facilitate tRNA^Lys3 ^annealing to genomic RNA *in vitro*, the annealing of primer tRNA onto the genomic RNA within HIV-1, murine leukemia virus, and avian retrovirus occurs independently of precursor protein processing [12-14]. However, while, tRNA^Lys3 ^is annealed efficiently in protease-negative HIV-1 (about 80% that found in wild-type virions), optimal placement on the viral genome to achieve efficient initiation of reverse transcription requires exposure of the viral genome to mature nucleocapsid protein [15]. In these protease-negative viruses, mutations in NC sequences within Gag inhibit tRNA^Lys3 ^annealing, while mutations in NC sequences within GagPol do not, indicating the importance of Gag NC sequences in the annealing [16]. *In vitro*, Gag has been reported to facilitate tRNA^Lys3 ^annealing to viral RNA as efficiently as mature NC [17].

Nevertheless, we will present evidence in this report that GagPol still plays an important role in tRNA^Lys3 ^annealing onto the viral RNA, independent of its role in the packaging of tRNA^Lys3 ^into the virion. We present data herein indicating that the RT connection domain, while non-essential for tRNA^Lys3 ^incorporation into virions, is required for tRNA^Lys3 ^annealing to the viral RNA genome

## Results

*The RT connection domain within GagPol is not required for tRNA^Lys ^incorporation into virions, but is required for the annealing of tRNA^Lys3 ^to the viral genome*.

**Figure 1:**
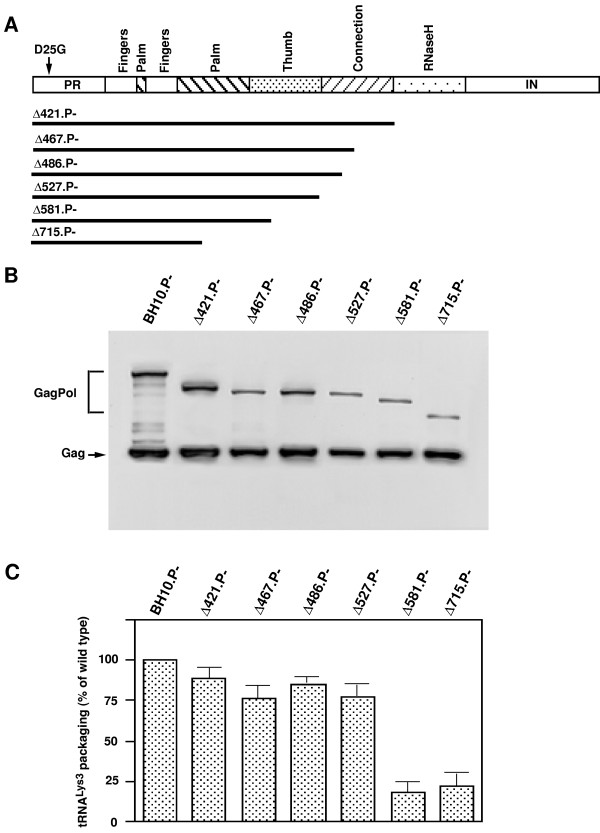
*The incorporation of GagPol and tRNA^Lys3^into wild-type and mutant HIV-1. ***A**. Schematic showing the deletions made in the Pol region of GagPol. Δ# designates the number of amino acid residues deleted from the C terminus of GagPol, and solid black lines represent the sequences not deleted. The RT sequence is divided into its known structural domains. The mutation D25G inactivates the viral protease. **B. **Western blots of viral lysates, probed with both anti-CA and anti-RT as previously described [18]. **C. **Incorporation of tRNA^Lys3 ^into wild-type and mutant virions. Dot blots of viral RNA were hybridized with probes specific for tRNA^Lys3 ^or genomic RNA, and the tRNA^Lys3^:genomic RNA ratios, normalized to BH10.P- were determined by phosphorimaging. The values are the means +/- standard deviations of experiments performed three or more times.

293T cells were transfected with protease-negative HIV-1 proviral DNA coding for either full length, protease-negative, GagPol (BH10.P-) or C-terminally deleted GagPol species. The different constructs are shown in Figure 1A, and are named according to the number of amino acids deleted from the C terminus of GagPol. Figure 1B shows Western blots of lysates of the viruses produced from the different transfections, probed with anti-CA, and shows that all forms of GagPol deletion mutants tested here are incorporated into the virion. Total viral RNA was isolated from these virions, and dot blots of this RNA were annealed with probes specific for either viral genomic RNA or tRNA^Lys3^, to determine the tRNA^Lys3^/genomic RNA in each viral variant. These results are shown graphically in Figure 1C, and support our previous results using COS7 cells [18], which indicate that tRNA^Lys ^incorporation into virions is not dramatically affected until GagPol sequences including the thumb domain of RT are deleted (Δ581 and Δ715).

**Figure 2:**
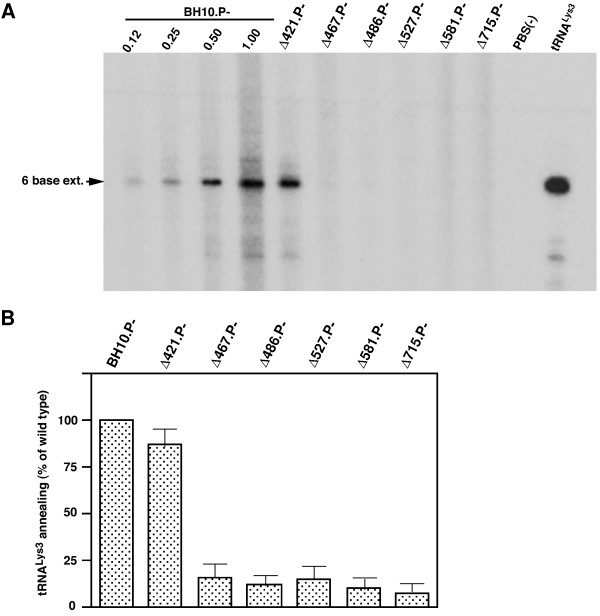
*tRNA^Lys3 ^annealing to viral genomic RNA. ***A. **Total viral RNA was used as the source of primer tRNA^Lys3^/viral RNA template in an *in vitro *reverse transcription reaction as described in Methods. Six base extended tRNA^Lys3 ^was resolved by 1D PAGE and quantitated by phosphorimaging. Each reaction used an equal amount of viral genomic RNA, as determined by hybridization with a genomic RNA-specific probe. **B. **Graphic presentation of 6 base-extended tRNA^Lys3^:genomic RNA ratios, normalized to BH10P-. The values are the means +/- standard deviations of experiments performed three or more times.

To measure the amount of tRNA^Lys3 ^annealed *in vivo *to the viral RNA genome, total viral RNA was used as the source of primer/template in an *in vitro *reverse transcription reaction, using exogenous HIV-1 RT, dCTP, dTTP, α-^32^P-dGTP, and ddATP. This assay measures the amount of extendable tRNA^Lys3 ^placed onto the viral genome. It is not known if all annealed tRNA^Lys3 ^is extendable. Since the sequence of the first six dNTP's incorporated is CTGCTA, annealed primer tRNA^Lys3 ^will be extended by 6 bases, and the extended tRNA^Lys3 ^can be resolved and detected by one dimensional polyacrylamide gel electrophoresis (1D PAGE). These results are shown in Figure 2A, and presented graphically in Figure 2B. The left side of panel A shows that there is a linear increase in the reverse transcription signal over an almost 10 fold change in the amount of BH10.P- viral genomic RNA used in the reaction. The data in the right side of panel A indicate that C-terminal deletions of GagPol extending into the connection domain result in an 85% or greater decrease in the initiation of reverse transcription. Thus, the data in Figures 1 and 2 indicate that deletions extending into the RT connection domain do not significantly effect tRNA^Lys ^incorporation, but do severely reduce the ability of tRNA^Lys3 ^to be functionally annealed to the viral RNA genome.

### Rescue of tRNA^Lys3 ^annealing by GagPol

**Figure 3:**
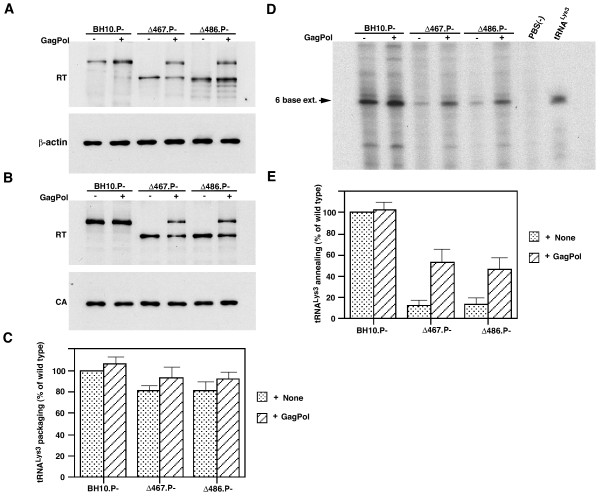
*Rescue by GagPol of tRNA^Lys3 ^annealing in mutant virions. *COS7 cells were transfected with either BH10P-, Δ467.P-, or Δ486.P-, and were also cotransfected with one of these plasmids and a plasmid coding for full-length GagPol (hGagPolΔFSΔPR). **A. **Western blots of cell lysates, probed with anti-RT or anti-β-actin. **B. **Western blots of viral lysates, probed with anti-RT and anti-CA. **C. **Incorporation of tRNA^Lys3 ^into wild-type and mutant virions. Dot blots of viral RNA were hybridized with probes specific for tRNA^Lys3 ^or genomic RNA, and the tRNA^Lys3^:genomic RNA ratios were determined by phosphorimaging. The values are the means +/- standard deviations of experiments performed three or more times. **D,E**. tRNA^Lys3 ^annealing in wild-type and mutant virions. tRNA^Lys3 ^annealing was measured as described in the Figure 2 legend. The values shown in E are the means +/- standard deviations of experiments performed three or more times.

As shown in Figure 3, this annealing defect can be rescued by coexpression of full-length GagPol. 293T cells were transfected with plasmids coding for BH10P-, Δ467, or Δ486, or cotransfected with either Δ467 or Δ486 and a plasmid coding for full-length GagPol. Western blots of cell lysates probed with anti-RT or anti-β-actin are shown in panel A, while Western blots of lysates of virus produced from these cells and probed with anti-RT and anti-CA are shown in panel B. These data indicate that both full length GagPol and the truncated GagPol are incorporated into the viruses with similar efficiencies. As previously indicated in Figure 1C, the mutant virions incorporate approximately 80–85% of the tRNA^Lys3 ^as BH10P-, but cotransfection of mutant DNA with DNA coding for GagPol gives a small increase in tRNA^Lys3 ^packaged to over 90% of BH10P- (Figure 3C).

As shown in panels D and E, cotransfection with GagPol also moderately rescues tRNA^Lys3 ^annealing in these mutant virions. Using equal amounts of total viral RNA as the source of primer/template in the *in vitro *RT assay, the ability of primer tRNA^Lys3 ^to be extended 6 deoxynucleotides is shown in panel D, which shows the extended 6 base product resolved by 1D PAGE. Quantitation of these bands by phosphorimaging is presented graphically in panel E. As previously shown (Figure 2), tRNA^Lys3 ^annealing is reduced to 12–15% that of BH10P-, but can be increased 4–5 fold by the additional presence of full-length GagPol. The fact that tRNA^Lys3 ^annealing is only rescued by GagPol to approximately 50–55% the level of that obtained when only wild-type GagPol is present may reflect the fact that in these rescue experiments, the viral population contains approximately equal amounts of wild-type and mutant GagPol (Figure 3B).

Attempts were also made to rescue tRNA^Lys3 ^annealing using mature RT fused to Vpr [19], but unlike full-length GagPol, the Vpr-RT was unable to rescue tRNA^Lys3 ^annealing in the mutant virions (data not shown).

## Discussion

*In vitro *studies of the interaction between purified RT and tRNA^Lys3 ^have indicated an interaction between the RT thumb domain and the tRNA [20-22]. *In vivo *studies also indicate an important role of the RT thumb domain in GagPol in tRNA^Lys3 ^viral packaging. tRNA^Lys3 ^incorporation into HIV-1 is not affected by deletion of the IN domain in GagPol, nor by further deletion of the RNaseH and connection domains in RT, but is severely inhibited by further deletion of the thumb domain as well [18]. Thus tRNA^Lys3 ^interacts with the RT thumb domain during incorporation into virions, and Gag nucleocapsid plays a role in promoting tRNA^Lys3 ^annealing to viral RNA [5-7], presumably through a denaturation of annealing RNA sequences.

What then is the role the RT connection domain sequence in GagPol in facilitating tRNA^Lys3 ^annealing? One possibility, suggested by *in vitro *studies, is that RT plays a direct role in tRNA^Lys3 ^annealing. Early work indicated that the *in vitro *annealing of primer tRNA^Trp ^to AMV genomic RNA was promoted by the addition of AMV reverse transcriptase [23]. In a later work, in which it was demonstrated that HIV-1 RT interacted with the D arm and TΨC loop of tRNA^Lys3^, HIV-1 RT was also shown facilitate the *in vitro *annealing of tRNA^Lys3 ^to the PBS sequence [24]. These *in vitro *works suggest that RT alone can directly promote tRNA^Lys3 ^annealing to viral RNA. Whether the RT sequences in GagPol can function similarly *in vivo *is not known.

Alternatively, the RT connection domain may undergo interactions with Gag that may result in placing the tRNA^Lys3 ^bound to the thumb domain in RT closer to either NC in Gag or to the genomic RNA that is bound to Gag NC. Recent work has indicated that that Pol sequences alone can bind to Gag p6 through the RT sequences in Pol [25]. Pol protein alone is sufficient for obtaining both tRNA^Lys ^incorporation into the virus and tRNA^Lys3 ^annealing to the viral genome at levels approximately 35% those achieved using full-length GagPol. Thus, in addition to the interactions which probably occur between Gag and homologous sequences in the Gag part of GagPol, the interaction of RT sequences in GagPol with Gag p6 could place the RT-bound tRNA^Lys3 ^closer to Gag NC sequences and viral RNA in the packaging complex. It remains to be determined which sequences within RT bind to Gag p6, but if it were those of the connection domain, this could explain how these sequences could promote tRNA^Lys3 ^annealing through altering the configuration of GagPol.

Thus, two separate RT domains (thumb and connection) appear to be involved, respectively, in the viral incorporation of tRNA^Lys3^, and its annealing to HIV-1 RNA. One also finds two separate domains in Gag involved in these same processes. Evidence has been presented supporting the role of lysyl-tRNA synthetase (LysRS) in targeting tRNA^Lys ^for viral incorporation, through a specific interaction of Gag capsid sequence with LysRS in a tRNA^Lys^/LysRS complex [26], while other evidence shows that Gag nucleocapsid sequence is involved in tRNA^Lys3 ^annealing [6,16,17]. It is not known if LysRS plays any direct role in tRNA^Lys3 ^annealing, and LysRS may be required to dissociate from tRNA^Lys3 ^so as to free this tRNA for annealing to the viral RNA.

## Methods

### Plasmid construction

BH10 and BH10P- are protease-positive and protease-negative strains of HIV-1, respectively [18]. All deletions mutants used here were derived from BH10.P-, and their construction has been previously described [18]. hGagPolΔFSΔPR was a gift from Y. Huang and G. Nabel [27]. It was constructed by deleting 5 thymidines in the frame shift site, and codes for GagPol. The codons have optimized for mammalian cell codon usage, which results in more efficient translation and protein production, and also makes nuclear export of these mRNAs Rev-independent through modification of the INS [27,28]. hGag-PolΔFSΔPR contain an inactive protease due to an R42G mutation in the active site.

### Production of wild type and mutant HIV-1 virus

Transfection of COS7 cells with wild type and proviral DNA was performed using the calcium phosphate method as previously described [29]. Briefly, virus were isolated from the cell culture medium 63 hours post-transfection. The supernatant was first centrifuged in a Beckman GS-6R rotor at 3000 rpm for 30 minutes, and the virus were then pelleted from the resulting supernatant by centrifuging in a Beckman Ti45 rotor at 35,000 rpm for one hour. The viral pellet was then purified by centrifugation at 26,500 rpm for 1 hour through 15% sucrose onto a 65% sucrose cushion, using a Beckman SW41 rotor.

### Protein Analysis

Viral particles were washed with 1X TNE and cellular or viral proteins were extracted with 1X RIPA buffer (10 mM Tris pH 7.4; 100 mM NaCI; 1% DOC; 0.1% SDS; 1%NP40; 2 mg/ml Aprotinin; 2 mg/ml Leupeptin; 1 mg/mlPepstatin A; 100 mg/ml PMSF). Western analysis was performed using 300 mg cellular protein or 10 μg viral protein, as determined by the Bradford assay [30]. The cellular and viral lysates were resolved by SDS-1D PAGE, followed by blotting onto nitrocellulose membranes (Gelman Sciences). Detection of protein on Western blots utilized monoclonal antibodies or antisera specifically reactive with viral capsid (mouse antibody, Intracel), viral reverse transcriptase (rabbit antibody), or β-actin (mouse antibody, Sigma Aldrich). Western blots were analyzed by enhanced chemiluminescence (ECL kit, Amersham Life Sciences) using goat anti-mouse or donkey anti-rabbit (Amersham Life Sciences) as a secondary antibody, and quantitated using UN-SCAN-IT gelTM automated digitizing system. The sizes of the detected protein bands were estimated using pre-stained high molecular weight protein markers (GIBCO/BRL).

### RNA Isolation and Analysis

Total viral RNA was extracted from viral pellets by the guanidinium isothiocyanate procedure [31], and dissolved in 5 mM Tris buffer, pH 7.5. To measure the incorporation of tRNA^Lys3 ^into virions, hybridization to dot-blots of viral RNA was carried out with DNA probes complementary to tRNA^Lys3 ^[1] or to genomic RNA [16]. To measure the amount of tRNA^Lys3 ^annealed to genomic RNA, tRNA^Lys3^-primed initiation of reverse transcription was measured using total viral RNA as the source of primer tRNA/template in an *in vitro *HIV-1 reverse transcription reaction, as previously described [32]. The sequence of the first 6 deoxynucleoside triphosphates incorporated is CTGCTA, and in the presence of dCTP, dGTP, dTTP, and ddATP, tRNA^Lys3 ^is extended by 6 bases, and this product can be resolved by 1D PAGE, and quantitated by phosphorimaging, as previously described [15].

## Authors' contributions

SC carried out the molecular genetic studies, assisted by MJ. LK conceived of the study, and participated in its design and coordination. All authors read and approved the final manuscript.
